# Peptidomics of potato protein hydrolysates: implications of post-translational modifications in food peptide structure and behaviour

**DOI:** 10.1098/rsos.172425

**Published:** 2018-07-11

**Authors:** Shixiang Yao, Chibuike C. Udenigwe

**Affiliations:** 1 College of Food Science, Southwest University, Chongqing 400715, People's Republic of China; 2 School of Nutrition Sciences, University of Ottawa, Ottawa, Ontario, Canada K1H 8L1; 3 Department of Chemistry and Biomolecular Sciences, University of Ottawa, Ottawa, Ontario, Canada K1N 6N5

**Keywords:** post-translational modification, bioactive peptides, food proteins, isoelectric point, hydrophobicity

## Abstract

Post-translational modifications (PTMs) often occur in proteins and play a regulatory role in protein function. There is an increasing interest in the bioactivity of food protein-derived peptides, but the occurrence of PTMs and their influence on food peptide structure and behaviour remain largely unknown. In this study, the shotgun-based peptidomics strategy was used to identify the occurrence of PTMs in peptides generated from potato protein hydrolysis using digestive proteases. Diverse PTMs were found in the potato peptides, including acetylation of lysine, N-terminal of proteins and peptides, C-terminal amidation, de-amidation of asparagine/glutamine, methylation and trimethylation, methionine oxidation and N-terminal pyro-glutamyl residue formation. The modifications may have been formed naturally or as a result of chemical reactions during isolation and enzymatic processing of the potato proteins. Most of the PTMs were calculated to decrease the isoelectric point and increase molecular hydrophobicity of the peptides, which will influence their bioactivity while also potentially altering their solubility in an aqueous environment. This is the first study to unravel that food-derived peptides can be widely modified by PTMs associated with notable changes in peptide chemical properties. The findings have broader implications on the bioavailability, biomolecular interactions and biological activities of food peptides.

## 1. Introduction

Specific food protein-derived peptide motifs, known as bioactive peptides, have regulatory functions in the body besides their nutritional functions [1]. The activities of bioactive peptides are attributed mostly to their low molecular size, chain length and chemical properties [2]. Among the latter, the global charge of peptides, positive or negative, is determined by the pH of their environment and will be completely lost at isoelectric points (pI). This will affect the charge-dependent bioactivity of peptides, including angiotensin I-converting enzyme (ACE) inhibitory and antimicrobial activities [3,4]. Furthermore, hydrophobicity of peptides can enhance their antihypertensive, antihyperlipidaemic and antioxidant activities, largely due to the hydrophobic interaction between peptides and their molecular targets, including proteins and lipids [5–7]. Additionally, peptide charge and hydrophobicity can also affect their bioavailability; for example, increased hydrophobicity will not only decrease aqueous solubility of peptides but also increase their bitterness property, thereby impeding their use in food application [8,9].

Peptide chemical property is determined by the nature of each of the constituent amino acid residues, especially their side chains [10]. Quantitative structure–activity relationship studies with food-derived bioactive peptides have focused on the relationships between native amino acid structures and peptide functions. Moreover, little consideration has been given to the susceptibility of side chains and N- or C-terminals of amino acids and proteins to chemical modifications, especially under food-processing conditions. For instance, post-translational modifications (PTMs) can occur in proteins and cause a change in their molecular size, charge and hydrophobicity, resulting in a significant change in protein function [11]. As PTMs play important roles in cells, a lot of attention has focused on the identification and functional elucidation of protein PTMs [12]. Currently, the well-resolved PTMs in cells include acetylation of protein N-terminal and lysine (Lys) residues [13], methionine (Met) oxidation [14], and methylation of Lys and arginine (Arg) residues [15]. The PTMs and their relevant biological functions have also been extensively studied in cells [11,16]. Moreover, we recently reported that food protein-derived peptides can be modified by Met oxidation and asparagine (Asn)/glutamine (Gln) de-amidation during isolation and enzymatic hydrolysis with pepsin [17]. In the present study, we further investigated, using the shotgun peptidomics workflow, the occurrence of a variety of specific PTMs in amino acid residues of peptides generated during the processing of potato proteins with gastrointestinal proteases, pepsin and pancreatin, and discussed the influence of such PTMs on the peptide structural properties and behaviour.

## 2. Material and methods

### 2.1. Preparation of the potato protein hydrolysate

Protein extraction from russet potato tubers was performed according to the previously reported protocol [18]. Briefly, potato tubers were fully homogenized with 5** **mM sodium bisulfite, and were then centrifuged at 3000*g* for 10** **min. The supernatant was adjusted to pH 5.0 (pI of patatin) using 1 M HCl. The sample was stored at room temperature for 15** **min, and was then centrifuged at 3000*g* for 20** **min. The pellet was collected and lyophilized.

The freeze-dried proteins were resuspended in water and hydrolysed with pepsin at an enzyme/substrate ratio of 1 : 100 (w/w) at 37°C and pH 2.0 for 1** **h, to mimic the gastric digestion conditions. The mixture was further digested with pancreatin at an enzyme/substrate ratio of 1 : 100 (w/w) at 40°C and pH 7.5 for 3** **h. Protein hydrolysates in both samples were heated at 90°C for 15** **min to inactivate the proteases. The mixtures were cooled to room temperature followed by centrifugation at 15 000*g* for 20** **min. The resulting supernatants were freeze-dried and dissolved in 0.1% formic acid prior to mass spectrometry (MS) analysis.

### 2.2. Liquid chromatography–tandem mass spectrometry analysis

For liquid chromatography–tandem mass spectrometry (LC-MS/MS) analysis, the peptide mixtures were separated by a 60-min gradient elution at a flow rate of 250 nl min^−1^ with an EASY-nLC integrated nano-HPLC system (Thermo Fisher, San Jose, CA, USA), which was directly interfaced with a quadrupole Orbitrap (Q-Exactive) mass spectrometer (Thermo Fisher, San Jose, CA, USA). The analytical column used was a PepMap RSLC EASY-Spray column (75** **µm × 50** **cm) packed with C18 resin (2** **µm). Eluted peptides from LC were injected into the Orbitrap Q-Exactive mass spectrometer, which was operated in the data-dependent acquisition mode using the Xcalibur software with a single full-scan spectrum (400–1500 *m/z*, 70 000 resolution) followed by 10 data-dependent MS/MS scans in the Orbitrap mass analyser.

### 2.3. Peptidomics data analysis

The PEAKS software (Bioinformatic Solutions Inc., Waterloo, ON, Canada) was used to perform the peptidomic analysis, including database search, peptide and PTM identification, as previously reported [17]. Both de novo sequencing and database search strategy were used by the PEAKS software for mass spectra analysis [19]. The protein database of potato (*Solanum tuberosum*) was retrieved from Uniprot (http://www.uniprot.org/). The database search parameters were set as follows: precursor mass tolerance, 10** **ppm; fragment ion mass tolerance, 0.02** **Da. Variable PTMs were set as follows: acetylation (protein N-terminal, peptide N-terminal, Lys), amidation (C-terminal), de-amidation (Asn/Gln), methylation (Cys/Asp/Glu/His/Ser/Thr/Lys/Arg/C-terminal), oxidation (Met), pyro-Glu (N-terminal Gln and Glu) and trimethylation (Lys/Arg), prior to PEAKS analysis. The false discovery rate (FDR) was calculated based on the target-decoy search strategy; the FDR value was set as 1.0% for peptide-spectrum matches to ensure reliable identification [20,21]. Ascore was employed by the PEAKS software to evaluate the reliability of PTM identification. Ascore calculates −10 × log_10_ P, where P indicates the likelihood that the peptide is identified by chance, and thus higher Ascore depicts more reliable identification [22]. The value of Ascore was set at greater than 20 to obtain reliable PTM sites.

### 2.4. Calculation of isoelectric point and hydrophobicity of peptides

To calculate the peptide chemical property, peptide sequences were analysed with Prot pi Peptide Tool (https://www.protpi.ch/Calculator/PeptideTool). Specific PTMs including Lys acetylation, N-terminal pyro-glutamylation and Asn de-amidation were set for the peptides, with ‘local’ modifications and the applicable PTM location. The default parameters were set to calculate the values of peptide pI and hydrophobicity. For pI values, peptide charge was calculated at pH 7.4 and pKa values were selected from Protein Modification Screening Tool. Retention coefficients were used to calculate the hydrophobicity of the peptides.

## 3. Results

### 3.1. Post-translational modifications in potato peptides revealed by peptidomics analysis

LC-MS/MS analysis of the potato protein hydrolysate gave a set of high-quality mass spectra, including 5128 MS1 spectra and 14 916 MS2 spectra. A total of 5133 peptide-spectrum matches were identified according to the data filter standard, and 3972 peptide sequences originating from 560 proteins were identified.

As shown in table 1, 125 acetylation sites were identified to occur at different locations, including protein N-terminal, which was predominant, followed by peptide N-terminal and Lys acetylation. Thirty-eight peptides were identified to be modified by C-terminal amidation, and over twice more peptides were identified to be de-amidated at either Asn or Gln residues. Forty-four methylation modifications were identified to occur at various amino acid residues, including Cys/Asp/Glu/His/Ser/Thr (16), Lys/Arg (9) and C-terminal (19). Furthermore, 194 sites of Met oxidation were identified, and 100 pyro-Glu modifications were found to occur mostly (91%) at N-terminal Gln, but also at N-terminal Glu residues. Lastly, 24 trimethylated sites were identified at the side chains of Lys/Arg residues of the peptides. A complete list of the identified potato peptides with PTMs has been provided in electronic supplementary material, table S1.

**Table 1.** Post-translational modifications (PTMs) found in peptides derived from potato protein hydrolysis with digestive proteases.

PTM	position	Δmass	no. peptides
acetylation	protein N-terminal	42.01	107
acetylation	N-terminal	42.01	12
acetylation	Lys	42.01	6
amidation	C-terminal	0.98	38
de-amidation	Gln/Asn	0.98	83
methylation	Cys/Asp/Glu/His/Ser/Thr	14.02	16
methylation	Lys/Arg	14.02	9
methylation	C-terminal	14.02	19
oxidation	Met	15.99	194
pyro-Glu	N-terminal Gln	17.03	91
pyro-Glu	N-terminal Glu	18.01	9
trimethylation	Lys/Arg	42.05	24

### 3.2. Post-translational modification, isoelectric point and hydrophobicity of peptides

Three unique PTMs, Lys acetylation, N-terminal pyro-Glu and Asn de-amidation, were selected for further evaluation of the effect of food peptide modification on their pI and hydrophobicity. It was apparent that the different PTMs exerted different effects on the peptide chemical properties (electronic supplementary material, table S1). Five randomly selected peptides from each PTM type are presented in table 2. Lys acetylation decreased the pI of the various peptides and had little influence on their hydrophobicity value. For example, the pI of AKDPVRVLVTGAAGQIGY decreased by 32% due to acetylation of Lys2, while the hydrophobicity remained the same. On the other hand, formation of pyro-Glu decreased the pI and increased the hydrophobicity of the peptides (table 2). For example, conversion of N-terminal Gln residue of QALKTYGRD to pyro-Glu led to a 35.7% increase in the hydrophobicity value, with a 13% decrease in the pI value of the peptide. Similarly, Asn de-amidation also exerted a substantial dual effect on the pI and hydrophobicity of the peptides. For DTNGKKLNPNSS, a single N3 (Asn3) de-amidation caused a decrease of the peptide pI by 30% and a twofold increase of its hydrophobicity (table 2).

**Table 2.** Effect of three PTMs on the chemical properties of representative peptides. PTMs, post-translational modifications; pI*, isoelectric point of unmodified peptide; pI, isoelectric point of modified peptide; H*_R_, relative hydrophobicity of unmodified peptide; H_R_, relative hydrophobicity of modified peptide.

sequence	PTMs	*Δ*mass	pI*	pI	*Δ*pI	H*_R_	*H*_R_	*ΔH*_R_
AKDPVRVLVTGAAGQIGY	acetylation (K2)	+42.0106	8.628	5.890	−2.738	32.33	32.33	0
NRQLSVLHPIHKLLHPHFRDTMNINA	acetylation (K12)	+42.0106	10.99	9.765	−1.228	36.87	36.87	0
TISKKLQ	acetylation (K5)	+42.0106	9.801	8.410	−1.391	10.11	10.11	0
VLAGRKTGGYVE	acetylation (K6)	+42.0106	8.470	6.107	−2.363	14.01	14.00	−0.01
KDRENGLF	acetylation (K1)	+42.0106	5.695	4.478	−1.217	17.49	17.42	−0.07
QAFRTIRDIEGNPLNKNSRY	pyro-Glu (Q1)	−17.0265	10.06	10.06	0	27.94	31.94	4
QAKEGRILLLKPTPSDIIY	pyro-Glu (Q1)	−17.0265	8.081	7.041	−1.040	37.15	41.15	4
QALDSQNNYLRVQE	pyro-Glu (Q1)	−17.0265	4.497	3.705	−0.792	21.86	25.90	4.04
QALEDDPFPFV	pyro-Glu (Q1)	−17.0265	3.552	3.552	0	35.43	39.44	4.01
QALKTYGRD	Pyro-Glu (Q1)	−17.0265	8.121	7.061	−1.060	11.19	15.19	4
AVGKVLPSLNGKLTGMA	de-amidation (N10)	+0.984	9.805	8.528	−1.277	30.62	34.70	4.1
DTNGKKLNPNSS	de-amidation (N3)	+0.984	8.586	5.994	−2.592	3.990	7.98	3.99
LEGQLQEVDNNKDAR	de-amidation (N10)	+0.984	4.311	4.100	−0.211	12.57	16.62	4.02
SVQGDPTNGLQGKHSNPA	de-amidation (N8)	+0.984	6.476	5.253	−1.223	9.650	13.65	4
NIGGNFKNGYPR	de-amidation (N8)	+0.984	9.805	8.359	−1.446	18.37	22.45	4.05

**Figure 1.**
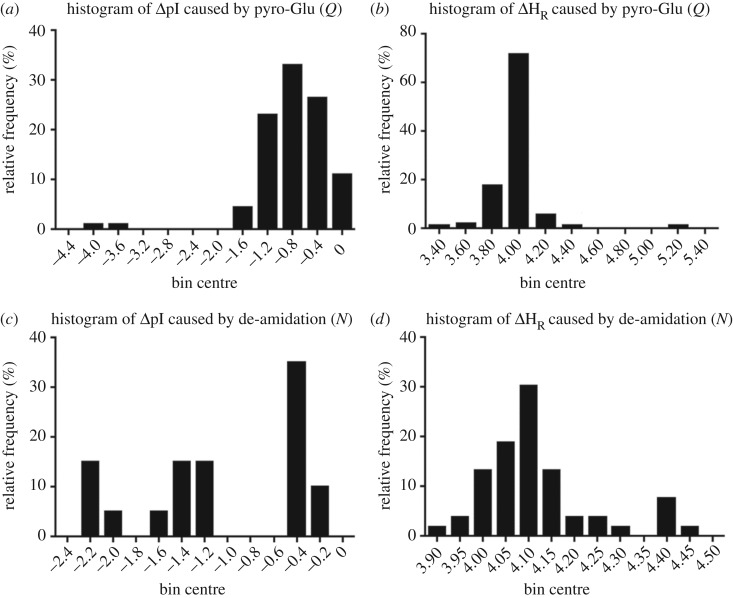
Histograms showing changes in peptide chemical property caused by PTMs. Effect of pyro-Glu formation (at N-terminal Gln) on (*a*) isoelectric point (pI) and (*b*) hydrophobicity (H_R_). Effect of Asn de-amidation on (*c*) pI and (*d*) H_R_. *Δ*pI, difference of pI value for modified peptide and that of unmodified peptide; *ΔH*_R_, difference of relative hydrophobicity value of modified peptide and that of the unmodified peptide.

To get a global picture of the PTM effect, all the peptides modified by Gln pyro-glutamylation and Asn de-amidation, which influenced both pI and hydrophobicity, were further evaluated. Figure 1 shows the histogram distributions. Pyro-Glu decreased the pI values, with approximately 82% of the modified peptides showing ΔpI between −0.2 and −1.4. The N-terminal cyclization also increased the hydrophobicity values, by factors of 3.9–4.1 in 71% of the pyro-Glu peptides. Similarly, Asn de-amidation increased the peptide hydrophobicity values across the board, but with a wider distribution when compared with the pyro-Glu data. De-amidation also decreased the peptide pI, with many peptides showing high ΔpI in the range of −1.2 to −2.4 (figure 1).

## 4. Discussion

PTMs are widely distributed in proteins where they play a critical role in regulating their biological functions [11]. However, there is a dearth of information on the occurrence and effect of PTMs on the property and function of food protein-derived peptides. In this study, the shotgun-based peptidomics strategy was used to systematically identify PTMs in peptides generated from potato protein hydrolysis with enzymes of the digestive tract. Some of the PTMs affect some bioactivity-related chemical properties of the food peptides.

To date, research on the functionality and bioactivity of food peptides focuses on their native amino acid sequence [2]. We thought that this can be quite different from the actual situation because of the propensity of amino acid residues to undergo chemical modifications. Moreover, it is common that proteins naturally undergo various PTMs. For instance, most mammalian proteins can be modified by one or more PTMs at some point, and more than 300 types of PTMs have been found to occur in the various proteins, either at the N- or C-terminal, or the amino acid side chains [11,23]. Similarly, PTMs can occur *in vitro* during food processing, such as protein extraction and enzymatic processing, due to chemical reactivity of several amino acid residues that may occur on heating and with other chemical species present in the mixture. To evaluate this possibility, we used a high-resolution mass spectrometer and an efficient mass spectra analysis software, which incorporates de novo sequencing and database strategy, to obtain reliable peptide identification [19]. Various PTMs were found to occur in the potato protein-derived peptides and a total of 608 modified peptides were identified, belonging to seven PTM types including acetylation, C-terminal amidation, de-amidation, methylation, oxidation, pyro-glutamylation and trimethylation. Owing to the principle of shotgun-based MS, only the most abundant peptides within the mixtures that eluted from LC to MS were selected for MS/MS analysis, which is critical for peptide identification. Peptides with PTMs are usually in low abundance compared to their unmodified counterparts, and thus are prone to being omitted in MS/MS analysis [24,25]. Hence, PTMs in the potato peptides could be far more abundant and diverse than the present study identified by shotgun-based peptidomics.

**Figure 2.**
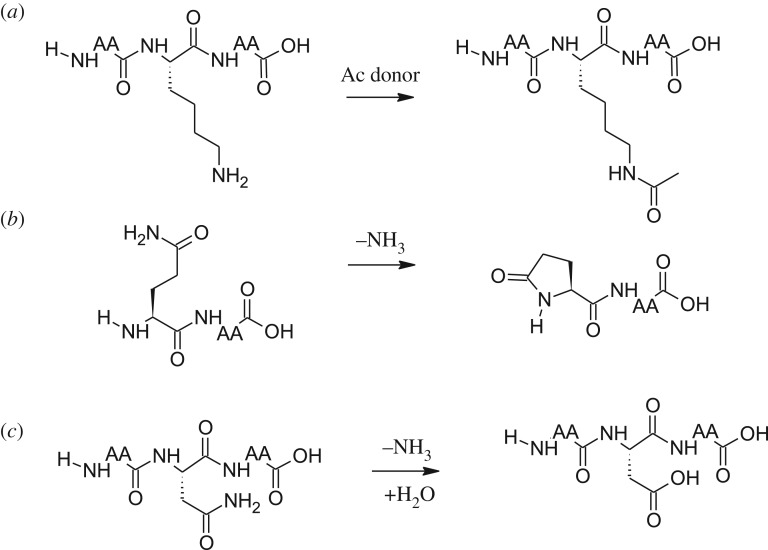
Reaction schemes for the formation of PTMs: (*a*) Lys acetylation, (*b*) pyro-glutamylation, and (*c*) Asn de-amidation in peptides; (*a*) decreased peptide pI values, whereas (*b*) and (*c*) both decreased pI and increased hydrophobicity of the peptides.

In principle, the PTMs identified in the peptides can be formed *in vivo* or *in vitro*. For instance, acetylation occurs in both prokaryotic and eukaryotic proteins [13,26] at the ϵ-amide group of Lys, as shown in figure 2. This results in a significant change in the chemical characters of the Lys residues, and has the potential to affect the function of various proteins. Lys acetylation occurs via either enzymatic or non-enzymatic pathways [27]; hence, processing is a plausible contributor to Lys acetylation in this study. Moreover, the protein N-terminal acetylation identified in the potato peptides is common in eukaryotic cells, and 30–80% eukaryotic proteins have been estimated to undergo this PTM, which is catalysed by N-terminal acetyltransferases [28,29]. It is interesting that we also identified 12 digested peptides with acetylated N-terminal residues, which is different from N-terminal acetylation on the precursor proteins. To the best of our knowledge, this is the first report to identify N-terminal acetylation of peptides generated from protein hydrolysis. This modification most probably occurred during processing via the reaction of N-terminal Lys of cleaved peptides and endogenous acetyl coenzyme A, because no acetyl donor was used as a reagent during processing.

Pyroglutamate is a cyclic amino acid found at the N-terminal of some proteins in cells. Pyro-Glu is formed via the rearrangement of N-terminal Glu or Gln residues, which could occur spontaneously and via a glutaminyl cyclase-dependent process, to form a γ-lactam ring structure [30], as shown in figure 2. Reaction rates of both synthetic pathways appear to be much faster with Gln residues [31]. Our results support this pattern, with the identification of over 10 times more pyro-Glu residues derived from N-terminal Gln-containing peptides than from N-terminal Glu peptides (table 1). Pyro-Glu residues can easily be formed where applicable under food-processing conditions, considering in this case that heat treatment is conventionally used to inactivate proteases after food protein hydrolysis. Moreover, Asn/Gln de-amidation, one of the most frequently occurring PTMs, is a non-enzymatic reaction, in which the amide group of the Asn/Gln side chain is converted into a carboxyl group (figure 2). Asn and Gln de-amidation occurs first by the formation of a succinimide intermediate product, which is then converted into aspartyl (or iso-aspartyl) and glutamyl (or γ-glutamyl) residues, respectively. De-amidation of amino acid residues can also occur *in vivo* and *in vitro*, at high alkaline and acidic pH conditions [32,33]. Therefore, it is plausible that the processing method used for protein hydrolysis (pH 2, pepsin) contributed to the 83 de-amidation sites identified in the potato peptides.

The most abundant PTM in our study was Met oxidation (table 1). This PTM is a prevalent modification that occurs in proteins *in vivo* or *in vitro* [34]. In human cells, oxidation of specific Met residues can modulate the function of proteins and signalling pathways, e.g. antibody function and immune response [34,35]. It is possible that the Met oxidation found in the potato peptides occurred endogenously, or during enzymatic processing of the proteins. Moreover, methylation and trimethylation also occurred in various amino acid residues of the peptides. Methylation usually takes place on Lys or Arg residues, which can be methylated once or more by lysine methyltransferases and arginine methyltransferases, respectively [36]. The most elucidated form of protein methylation in cells occurs at the Lys/Arg residues of histones, which is a critical epigenetic regulator of gene expression [37]. As there is no evidence to support the occurrence of methylation via non-enzymatic pathways, we suggest that the methylation and trimethylation observed in this study may have occurred endogenously instead of during isolation and enzymatic processing of the proteins.

The C-terminal carboxyl group of proteins is usually free from covalent modifications, with a few exceptions such as amidation. C-terminal amidation usually occurs in peptide hormones via enzymatic catalysis [38], and approximately 50% of all neuropeptide hormones are estimated to exhibit such PTM [39]. Non-enzymatic processes could also amidate the C-terminal of peptides [40]. Taken together, the processing condition used to produce the peptides is one plausible contributor to formation of the PTMs identified in this study. With significant structural alterations, the PTMs can influence the behaviour of the peptides in complex biological matrices.

Three identified PTMs including Lys acetylation, pyro-Glu formation and de-amidation were selected to elucidate the influence of PTMs on some chemical properties of the food peptides. As shown in table 2 and figure 1, the different PTMs have different effects on the peptide pI and hydrophobicity. For instance, Lys acetylation was found to decrease the pI values, whereas pyro-Glu and de-amidation decreased the pI and increased the hydrophobicity. Peptide charge, determined by the pH of its environment, has been shown to influence the bioactivities of food peptides. Positively charged Lys and Arg residues contribute to ACE inhibitory activity [4] by enhancing interaction with anionic allosteric binding sites of the enzyme. Likewise, with a clearly defined mechanism, cationic antimicrobial peptides rely on their global positive charge to exhibit their activity. As Lys acetylation and Asn de-amidation alter the pI and possibly global peptide charge, it is reasonable to expect that various bioactivities of the peptides will change because of the modifications. Likewise, peptide hydrophobicity is often associated with many of their biological activities, including ACE inhibition, antimicrobial, antioxidant and bile acid-sequestering activities [1,2,5–7,41–43], and these bioactivities can potentially be altered considering the magnitude of the structural changes induced by de-amidation and pyro-glutamylation. Other hydrophobicity-dependent properties of peptides, such as solubility, also need to be considered. In fact, peptide aggregates formed during protein hydrolysis [44] may be partly because of the increased hydrophobicity induced by PTMs. Pyro-Glu significantly enhances peptide hydrophobicity due to formation of the γ-lactam ring at the N-terminal. Previously, pyro-Glu residues were detected in hydrolysed wheat gluten [45], and its formation has been associated with decreased solubility and the aggregation of amyloid *β* peptide in neurodegenerative diseases [46]. Finally, peptide hydrophobicity is important for their transepithelial transportation [47] and bitter-tasting properties [8,9]. Peptide modification has been suggested to be a critical factor to consider when including peptides as active components of functional food products [48].

## 5. Conclusion

The shotgun-based peptidomics strategy was used in this study to analyse potato protein-derived peptides, and the results demonstrate the occurrence of several PTMs, including acetylation, methylation, Met oxidation, pyro-Glu formation, de-amidation and amidation. Chemical reactions during isolation and enzymatic processing of the proteins are plausible factors responsible for the PTM formation. Some of the PTMs affect the chemical properties of peptides, including pI and hydrophobicity, and may also influence their interaction with other molecules in food and physiological matrices. De-amidation and pyro-glutamylation increased the hydrophobicity of peptides, which can affect their binding to physiological targets, or decrease their solubility, thereby impeding their bioavailability.

## Supplementary Material

Complete data and PTMs identified in detected peptides present in the potato protein hydrolysate

## Data accessibility

Electronic supplementary material, table S1, has been uploaded as the dataset supporting this article.
